# Antibody Therapeutics in Oncology

**Published:** 2016-02-01

**Authors:** Erik D Wold, Vaughn V Smider, Brunhilde H Felding

**Affiliations:** 1 Department of Chemical Physiology, The Scripps Research Institute, 10550 N Torrey Pines Road, La Jolla, CA 92037, USA; 2 Department of Cell and Molecular Biology, The Scripps Research Institute, 10550 N Torrey Pines Road, La Jolla, CA 92037, USA; 3 The Scripps Research Institute, 10550 N Torrey Pines Road, Mail drop MEM 150, La Jolla, CA 92037, USA

**Keywords:** Cancer, Metastasis, Metabolism pathway, Carcinogenesis

## Abstract

One of the newer classes of targeted cancer therapeutics is monoclonal antibodies. Monoclonal antibody therapeutics are a successful and rapidly expanding drug class due to their high specificity, activity, favourable pharmacokinetics, and standardized manufacturing processes. Antibodies are capable of recruiting the immune system to attack cancer cells through complement-dependent cytotoxicity or antibody dependent cellular cytotoxicity. In an ideal scenario the initial tumor cell destruction induced by administration of a therapeutic antibody can result in uptake of tumor associated antigens by antigen-presenting cells, establishing a prolonged memory effect. Mechanisms of direct tumor cell killing by antibodies include antibody recognition of cell surface bound enzymes to neutralize enzyme activity and signaling, or induction of receptor agonist or antagonist activity. Both approaches result in cellular apoptosis. In another and very direct approach, antibodies are used to deliver drugs to target cells and cause cell death. Such antibody drug conjugates (ADCs) direct cytotoxic compounds to tumor cells, after selective binding to cell surface antigens, internalization, and intracellular drug release. Efficacy and safety of ADCs for cancer therapy has recently been greatly advanced based on innovative approaches for site-specific drug conjugation to the antibody structure. This technology enabled rational optimization of function and pharmacokinetics of the resulting conjugates, and is now beginning to yield therapeutics with defined, uniform molecular characteristics, and unprecedented promise to advance cancer treatment.

## Introduction

Cancer, in its most basic definition, is a class of diseases characterized by abnormal cell growth. Cancer affects essentially all macro-organisms, and paleo-pathologic findings suggest that cancer has existed since long before humans [1]. The earliest known recorded case of cancer, a breast cancer, was found in an ancient Egyptian medical text (the Edwin Smith Papyrus written circa 3000 B.C.), which described the ailment as a grave disease with no treatment [2]. Since its initial identification 5000 years ago, the treatments for cancer, while varying wildly in the specific methods and outcomes, have remained quite consistent. Essentially every medical procedure for cancer throughout history employed surgical and chemotherapeutic intervention, and recognized that early diagnosis and intervention were necessary for successful treatment [3-6]. The causes and progression of cancer, however, remained essentially a complete mystery until around mid-19th century.

Tumor metastasis was originally described by the 1st century Roman physician Anulus Celsus, in his De Medicina encyclopedia of medicine [1]. Celsus described secondary tumours and recurrences of breast cancer in the armpits, and noted that in advanced cases death may be caused by spreading of the disease to distant organs [7]. It took another 2000 years, however, to develop the modern model for cancer progression and metastasis. Campbell De Morgan, after 34 years of clinical study, developed his model for the “focal origin of cancer” [8]. In a series of publications between 1872 and 1874, De Morgan described cancer as a disease that arose locally before spreading, first to the lymph nodes and then throughout the body [9-11]. Furthermore, it was around this time that chemical carcinogens were first discovered in association with occupation risk of lung cancer. Miners, and other industrial workers, where shown to have significantly higher rates of lung cancer, which researchers eventually attributed to the inhalation of arsenic, bismuth and other chemicals in the form of mine dust [12]. In the following decades, the discovery of both x-rays and radioactivity revolutionized the treatment and diagnosis of cancer. Importantly, both of these types of ionizing radiation were eventually also identified as another significant source of carcinogenesis [12].

In the decades following these discoveries, more cancer research took place than during the prior centuries combined. First and foremost, the hypotheses that cancer was caused by demons, moral and religious deviations, and humoral imbalances were largely disabused by this point [13]. The discoveries of the mid to late 19^th^ century coupled with the advent of *in vitro* and *in vivo* cancer models resulted in a great advancement in the field of cancer research [5,13,14]. During this time, histopathological staging of tumours was first introduced, a number of new cancers and carcinogens were discovered, and *in vitro* and *in vivo* techniques enabled early investigation in carcinogenesis and the biology and biochemistry of cancer cells [13,15,16]. The connection between genetics and cancer, which was first suggested in the mid to late 19th century, was not discovered until the early 20th century with the advent of *in vivo* cancer biology and genetically controlled animal strains [8,13,17].

One of the most important discoveries of this time was made by German biochemist Otto Warburg in 1924 [18]. He discovered that cancer cells metabolize glucose in a manner that is distinct from the main energy metabolism pathway used by normal cells and tissues. While normal cells derive energy primarily from oxidative phosphorylation through mitochondrial respiration, cancer cells use glycolysis, even in the presence of sufficient oxygen to support mitochondrial oxidative phosphorylation [19-24]. This discovery is the basis for positron emission tomography (PET) imaging of tumours, an invaluable tool in modern cancer diagnosis and treatment, based on the differential uptake of 18F labelled glucose derivatives by cancer cells compared to normal cells [25-29].

Warburg went on to hypothesize that this phenomenon was not just a feature of cellular transformation, but that cancer was caused by mitochondrial damage, resulting in lower oxidative phosphorylation and higher levels of glycolysis [30]. Since then, the cancer research community has largely discredited this hypothesis, stating that the metabolic changes observed in cancer are a result of cellular transformation with the anaerobic tumor microenvironment selecting for increased glycolysis. Down-regulation of oxidative phosphorylation in response to oncogene activation was considered an advantage for tumor cells that could foster adaptation to hypoxic conditions [31-33]. However, Warburg’s hypothesis may have been more appropriate than initially given credit for. During the current renaissance of research into cancer metabolism, there have been a number of studies showing that damaged mitochondria directly facilitate a more aggressive cancer phenotype, and that normalization of mitochondrial function in cancer cells can reduce tumorigenesis and metastatic activity [34-43]. Thus, while mitochondrial dysfunction in conjunction with oncogenic events may not be the exclusive cause of all cancers, as Warburg initially hypothesized; mitochondrial functionality is certainly intimately involved in tumorigenesis and cancer progression [44-46].

The era of the late 19th and early 20th century also provided the very first examples of cancer immunotherapy, another area of cancer research that is currently undergoing a renaissance of research [8,12]. Clinical reports in the late 19th century described occasional spontaneous remission of various cancers when patients co-presented with infectious diseases, notably erysipelas [47]. This phenomenon prompted investigation by William B. Cooley into the infection of cancer patients with various infectious agents, e.g. *Serratia marcescens* or *Streptococcus pyogenes*, or administering extracts of these bacteria, in attempt to induce remission [48,49]. The idea behind Cooley’s toxin therapy is that the administered infectious agent or toxin would result in systemic immune activation with a desired side effect of tumor cell destruction by the activated immune system [50,51]. Even though the results and methods gained significant criticism over the years, Coley claimed great success, reporting an estimated 80% 5-year survival rate for diseases with no other treatment options [52-54]. Despite the literally unbelievable success rate, and the eventual discontinuation of Coley’s toxin cancer treatment, this approach was the first example of attempting to artificially induce an immune response as a cancer treatment; the basis for many of today’s promising cancer therapies.

Coinciding with the discontinuation of Coley’s toxin cancer treatments in the 1950s, was the discovery of tumor associated antigens: the next great advancement in the field of cancer immunotherapy [8,55]. The discovery of tumor associated antigens resulted in a massive amount of research over the following decades to identify new tumor associated antigens and develop therapies to take advantage of these new discoveries [56-69]. Unfortunately, efforts towards the development of cancer vaccines, and other immune based cancer therapies over this time period were largely ineffective [8,55,70]. However, the efforts towards the research of tumor associated antigens laid the groundwork for modern monoclonal antibody therapy and other immunotherapies for cancer. Concurrent with this research in cancer immunotherapy, research into carcinogens, tumorigenesis, diagnostic techniques, chemotherapeutics, radiation therapy, cancer classification, and surgical procedures all advanced by leaps and bounds. However, despite the advances in detection, diagnosis, and treatments of cancer, the overall mortality rate remained high, and relatively unchanged from previous generations [13,55].

Despite discovery of cancer as a deadly disease thousands of years ago, characterization of the disease progression, and accumulating knowledge on causes over the course of the last few hundred years, modern cancer treatment faces many of the same issues today as it has throughout history [1,3,4,12,13,55]. Treatment today generally follows the same scheme as it has since humans began attempting to treat this disease: Surgical intervention (if possible) to remove any primary and secondary tumors, and chemotherapeutic intervention to treat inoperable tumours and lesions to halt or prevent disease progression. Importantly, the issue of cancer metastasis still plagues clinicians and patients, without break-through advances toward truly effective therapies that can achieve a cure for patients with advanced cancer. The majority of deaths from cancer are caused not by the primary tumor, but by secondary growths that result from tumor invasion and metastasis, and advanced cancers still have a very poor prognosis [71].

Through advances in research, diagnostic, and surgical approaches, oncologists now have refined, clinically validated chemotherapeutics, combination treatments, and other cancer therapeutics making successful intervention much more common. Cancer survival has steadily increased in Western countries for the last 30 years [72,73]. This can be explained by a better understanding of cancer biology and disease progression, more advanced *in vivo* and *in vitro* cancer models, the sequencing of the human genome resulting in a clearer picture of the genetic contributions to cancer, an understanding of onco-genetics, the development of new and more accurate cancer screening techniques, and new, more targeted cancer therapeutics [74-84].

One of the newer classes of targeted cancer therapeutics is monoclonal antibodies. Monoclonal antibody (mAb) therapeutics are a successful and rapidly expanding drug class due to their high specificity, activity, favourable pharmacokinetics, and standardized manufacturing processes [85-94]. The success of monoclonal antibody therapeutics is built on the back of the aforementioned investigation into cancer immunology from the 1950s to the 1970s. Since the discovery of tumor specific antigens, researches attempted to use antibodies as therapeutic agents. Originally, researchers attempted to immunize an animal with cancer cells and then administer serum or polyclonal antibodies in attempt to confer passive immunization [55,62,95]. However, immunogenicity of the serum and/or antibodies, coupled with the irreproducibility of the animal immunizations made this therapy impractical and ineffective [55,62,95]. The development of hybridoma technology in 1975 re-invigorated researches into antibody based cancer therapeutics [96,97]. For the first time, monoclonal antibodies could be isolated and produced. Thus, individual antibody sequences could be cloned and screened for maximal efficacy instead of relying on the unpredictable and often irreproducible process of passive immunization with serum.

After initial issues with the immunogenicity of murine mAbs, and subsequent development of chimeric and humanized antibodies, mAbs have become some of the most successful and efficacious drugs of the last few decades [94,98,99]. There are currently 36 FDA approved monoclonal antibodies for the treatments of various diseases, with 17 for the treatment of cancer. While there are 5 classes of antibodies (immunoglobulin’s) in humans (IgG, IgA, IgE, IgD, and IgM), every currently clinically approved mAb therapeutic is an IgG (the immunoglobulin responsible for antibody-based immunity against pathogens) [100-103]. Assuming the availability of targetable and tumor specific antigen, antibody therapeutics are an ideal cancer drug.

Antibodies (IgGs in particular) are capable of recruiting the immune system to attack whatever they bind. The binding of 2 or more IgG1 molecules to the cancer cell surface results in binding of the C1q protein of the complement system which initiates activation of the complement cascade [104]. Activation of the complement system results in the formation of the membrane attack complex which causes membrane pore development and subsequent cell lysis. Complement activated cell lysis can additionally result in recruitment and activation of certain immune effector cells (macrophages, neutrophils, eosinophils etc.) [104,105]. This process is known as complement-dependent cytotoxicity (CDC). Furthermore, bound IgGs can recruit and activate immune cells directly via Fcγ receptor binding. Natural killer (NK) cells, macrophages, dendritic cells, neutrophils, eosinophils, and other immune cells all express various forms of the Fcγ receptor, enabling them to be recruited and activated to induce antibody dependent cellular cytotoxicity (ADCC) and antibody dependent cellular phagocytosis (ADCP) [106,107]. Moreover, in an ideal scenario the initial tumor cell destruction induced by administration of a therapeutic antibody can result in uptake of tumor associated antigens by antigen-presenting cells establishing a prolonged memory effect [96].

Importantly, many mAb therapeutics have intrinsic anti-cancer activity and exerts their effects without the need of immune activation. For example, several tumor associated antigens are growth factor receptors that are overexpressed at the tumor cell surface, and can be one of the driving forces of unregulated cellular growth as well as promote insensitivity and resistance to chemotherapeutic agents [108]. mAb therapeutics that bind such growth factor receptors are often capable of disrupting ligand binding or receptor signaling, which can potentially inhibit cell proliferation and re-sensitize tumor cells to chemotherapeutics [94,96,109].

Some of the first cell surface receptors to be targeted in this fashion are the EGFR family receptors [108]. Antibodies that target these receptors are among the most potent inhibitors of signal transduction. The specific mode of action of these antibodies can vary. Therapeutically used antibodies cetuximab and panitumumab physically block the interaction between the EGF receptor and its ligand via steric hindrance. This prevents the receptor from assuming the conformation required for dimerization and subsequent cell signaling [110-112]. Others, such as pertuzumab and trastuzumab which target the EGFR family member Her2 do not inhibit ligand binding but disrupt the ability for their target receptors to heterodimerize, thereby preventing receptor signaling [113,114]. Essentially every clinically effective, unconjugated mAb disrupts cell signaling of an overexpressed, proliferation-driving cell surface receptor, as opposed to relying strictly on antibody effector function for activity [96]. However, the efficacy of these antibodies is fully explained by a combination of immune activation and the antibody’s intrinsic ability to inhibit growth factor signaling [112,115,116].

**Figure 1:**
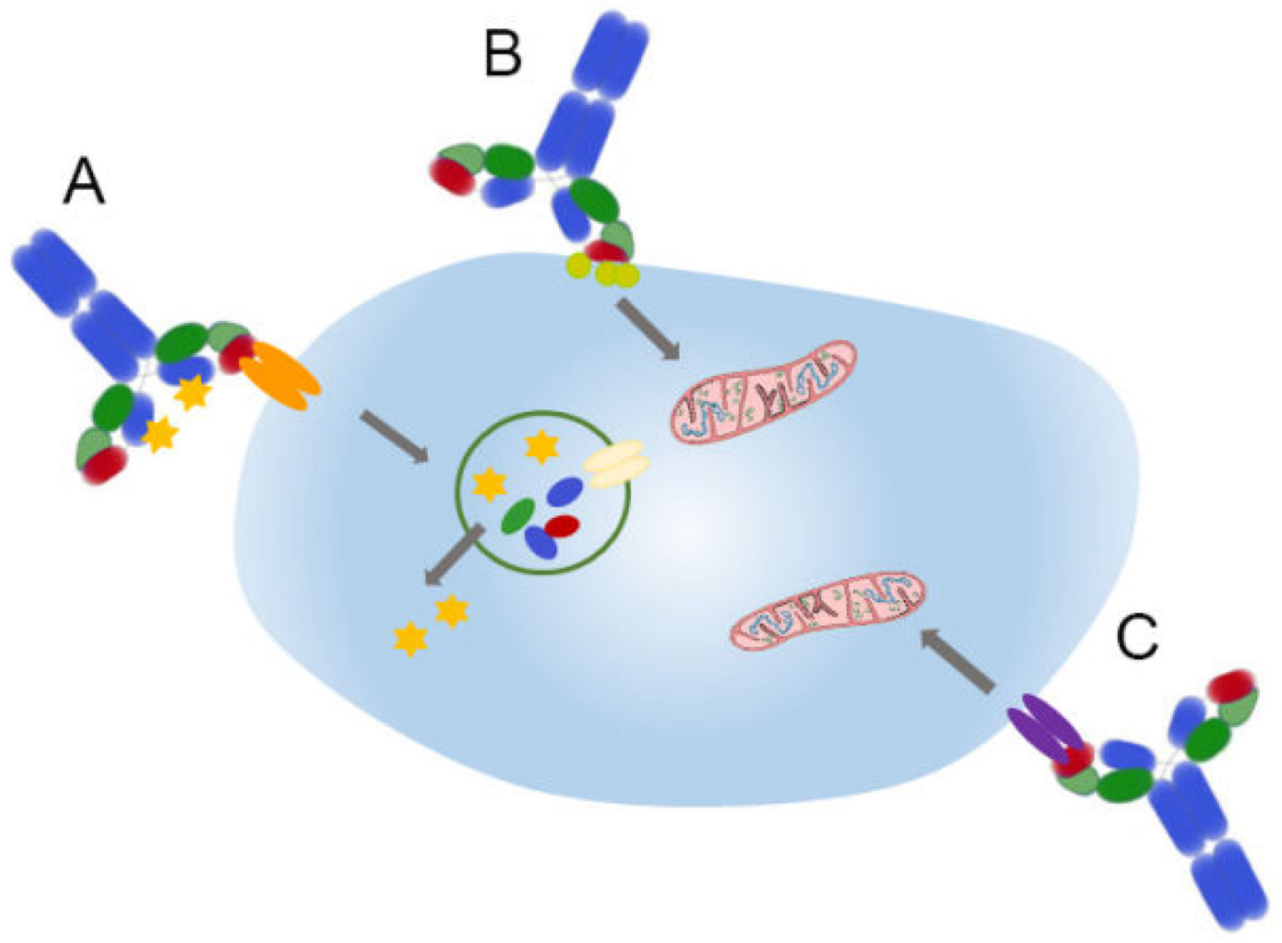
Mechanisms of direct tumor cell killing by antibodies. A: Antibody drug conjugates deliver cytotoxic drugs after selective binding to tumor cell surface antigens, internalization, and intracellular drug release; B: Antibodies against surface bound enzymes can neutralize enzyme activity and signaling, leading to apoptosis (symbolized by the mitochondrion); C: Antibodies can induce receptor agonist or antagonist activity and thereby cause apoptosis.

Generally, mechanisms of direct tumor cell killing by antibodies (Figure 1) may include antibody recognition of cell surface bound enzymes to neutralize enzyme activity and signaling. In other cases, antibodies can induce receptor agonist or antagonist activity. The result of both approaches is induction of cellular apoptosis. In another and very direct approach, antibodies are used to deliver drugs to target cells and cause cell death. Such antibody drug conjugates (ADCs) direct cytotoxic compounds to tumor cells after selective binding to cell surface antigens, internalization, and intracellular drug release. In this way, ADCs take advantage of the desirable specificity and pharmacokinetics of immunoglobulins as a means to deliver highly cytotoxic drug payloads [117,118]. This is done by conjugating cytotoxic drugs onto an antibody, so when the antibody binds its target cell and is subsequently internalized, the toxic drug is released and able to kill the cell [119]. ADCs offer significant promise as therapeutics because they are able to deliver highly toxic drugs very specifically to the tumor cells. Thus, untargeted toxicities associated with the use of free drugs are greatly reduced, and poor therapeutic indices associated with conventional chemotherapies can be significantly improved [120]. Many biotech and pharmaceutical companies are researching and pursuing ADCs as potential cancer therapeutics. There are currently over 30 ADC drugs in clinical trials for cancer therapy, in addition to two FDA approved ADCs (Kadcyla and Adcetris) being administered to patients [120-122]. Kadcyla is an ADC of trastuzumab and emtansine, the tubulin binding drug DM1 that potently blocks cell proliferation. This ADC is approved for breast cancer patients with Her2 positive tumours after prior treatment with trastuzumab alone and a taxane. Adcetris is an ADC of anti-CD30 and the highly toxic anti-mitotic agent auristatin E. Adcetris is being used for treatment of classical Hodgkin lymphoma and systemic anaplastic large cell lymphoma after other chemotherapies have failed.

There are distinct advantages to ADCs over unconjugated mAbs. Unconjugated mAbs, while they can induce an immune response and often disrupt cancer cell signaling, tend to require combination therapy with conventional chemotherapeutics to be efficacious [123,124]. Additionally, in pre-clinical research and in clinical trials the efficacy of ADCs was shown to be significantly better than unconjugated mAbs (or conventional chemotherapy) [119,125-127]. Importantly, the high specificity of the drug delivery mechanism allows for the application of drugs that would be otherwise too toxic to administer to a cancer patient [125,128-130]. Despite the use of extremely cytotoxic drugs, ADCs have proven to have toxicity profiles better than conventional chemotherapeutics [127,131,132].

The primary drawback to ADCs is their inherent complexity. Each ADC is comprised of at least 3 components: the antibody, a linker, and the drug. Each of these components can be varied, and the efficacy and safety profile of the resulting therapeutic can change drastically [128,133]. An additional confounding factor with the production of ADCs is using the commonly utilized conjugation methods of lysine amidation, or maleimide coupling of cysteines from reduced interchain disulfide bonds, resulting in a heterogeneous mixture of both location and number of conjugates per antibody molecule (usually resulting in a mixture of products comprising between 0 and 8 distinct conjugates) [134-136]. This can negatively affect both the toxicity of the ADC as well as its binding affinity [137-139]. An example is gemtuzumab ozogamicin, a previously FDA approved ADC consisting of anti-CD33 and a calicheamicin as a potent anti-tumor antibiotic. This ADC, tried against acute myelogenous leukemia, used random lysine conjugation and had to be pulled from the market because of toxicity and lack of efficacy [140].

A critical current area of research looking to improve upon some the issues plaguing ADC production and clinical use, is site-specific conjugation. Site-specific conjugation allows for control over the conjugate:antibody stoichiometry and the location of conjugation, which enables the generation of a homogenous product [140-142]. There are three primary methods to accomplish site specific conjugation: engineered cysteine residues, bio-orthogonal reaction with unnatural amino acids, and through enzymatic conjugation with glycotransferases [139,141-143]. Importantly, it has been recently shown that the site location, and conjugate:antibody stoichiometry can have a profound impact on conjugate stability and therapeutic activity of ADCs [144,145]. Thus, use of site-specific conjugation methods allows for optimization of therapeutic activity and toxicity profile of an ADC that would have been otherwise impossible with conventional conjugation chemistries.

Therapeutic antibody-drug conjugates, however, are not the only clinically relevant application of immunoconjugation. Radio-immunconjugates have been used in the treatment and diagnosis of various cancers [146-148]. Oligonucleotide-antibody conjugates have been used in highly sensitive immuno-PCR for the detection of circulating tumor cells (CTCs) or other rare cells, in the generation of highly sensitive antibody arrays, and for the generation of multimeric antibody constructs [149-153]. Bi-specific antibody constructs have been produced via conjugation to either gain synergistic activity against multiple tumor associated antigens, or to recruit and activate cytotoxic T-cells for cancer therapy [154-161]. Antibody conjugation has also been used to circumvent the often procedurally intensive process of antibody selection, by using the conjugated molecule for the specificity, and rely on the antibody scaffold to provide the desired effector function and pharmacokinetics [162-169].

Due to antibody specificity and target selectivity, no single antibody or antibody conjugate will be broadly applicable to cancer treatment. Thus, in order to exploit the extremely high selectivity of therapeutic antibodies, tumor associated antigens must be identified and their exclusive expression on tumours has to be validated to avoid off-target toxicity [170-175]. This is particularly important for ADCs, as these can be extremely effective at eliminating target cells identified by the antibody moiety. Unless a ubiquitous tumor associated antigen is discovered, each different type of cancer requires the development of an antibody specific to cell surface antigens expressed on that cancer. This requirement applies to each individual disease type. For example, breast cancer is a highly heterogeneous disease, with no less than 5 distinct molecular subtypes [176-180]. Only the HER2-enriched subtype currently has an antibody therapeutic as an available treatment (though ER+ breast cancer relies on anti-hormone therapy targeting estrogen as a disease-driving ligand) [181-186]. Breast tumours that do not express the estrogen receptor, the progesterone receptor, and are HER2-negative, are referred to as “triple-negative” which accounts for approximately 15%-20% of diagnosed breast cancers [178,187,188]. Triple negative breast cancer is generally the most aggressive subtype, has a particularly poor outcome, and there are no antibody therapeutics or targeted therapies available to treat this subtype of breast cancer [178,187,189-192]. Considering the need for targeted treatments of otherwise hard-to-manage and particularly aggressive types of cancer in general, identification of antigens specific for those tumor cells would revolutionize cancer therapy.

While there are many techniques for the discovery of antibodies against important disease-associated antigens, finding antibodies that bind defined functional epitopes with high specificity and affinity is not trivial. This is particularly true if one attempts to identify antibodies against an unknown target protein on a target cell type or cell line. Classical hybridoma mAb development approaches require animal immunization, B-cell fusion, clonal screening, and antibody gene cloning and sequencing, before a hybridoma derived mAb can begin preclinical testing as a potential therapeutic [97,193,194]. Additionally, many hybridoma derived mAbs will have to be humanized before clinical application [195]. Antibody display based approaches circumvent many of the issues associated with animal immunization and hybridoma development. Antibody display allows for the direct selection of fully human antibodies from recombinant antibody libraries, and the display systems are designed such that much of the cell and molecular biology associated with hybridoma development is unnecessary [196]. This significant improvement over classical mAb development approaches can be enhanced by smartly designed selection strategies, multiple and diversified rounds of selection, and clonal antibody screening [196-198]. Identification of antibodies with function blocking properties, or selection of antibodies that merely recognize epitopes unique to the target tumor cell type with high selectivity, should enable specific development of targeted antibody therapies that can effectively interfere with cancer progression, eliminate cancer recurrence, or prevent spreading of particularly aggressive cancer cell types. Deep analysis of antibody-antigen relationships, antigen distribution, and identification of patient target groups will lead to the development of novel targeted therapies for many cancer types that urgently require effective treatment approaches.
